# Elevated circulating BACE2 captures chronic glycemic burden and enhances the clinical identification of type 2 diabetes

**DOI:** 10.1016/j.isci.2026.115988

**Published:** 2026-05-22

**Authors:** Bo Li, Zhixing Luo, Zhongyu Chen, Han Wang, Shiqiao Zhao

**Affiliations:** 1 Chongqing Key Laboratory of Sichuan-Chongqing Co-construction for Diagnosis and Treatment of Infectious Diseases Integrated Traditional Chinese and Western Medicine, Chongqing 400016, China; 2 Department of Clinical Laboratory, Chongqing Hospital of Traditional Chinese Medicine, Chongqing 400016, China; 3 Department of Clinical Laboratory, Wulong District People’s Hospital, Chongqing 408500, China; 4 Laboratory for Clinical Diagnostic and Translational Research of Children’s Hospital of Chongqing Medical University, National Clinical Research Center for Child Health and Disorders, Ministry of Education Key Laboratory of Child Development and Disorders, Chongqing Key Laboratory of Pediatric Metabolism and Inflammatory Diseases, Chongqing 400014, China

**Keywords:** health sciences, medicine, medical specialty, endocrinology

## Abstract

Although β-site amyloid precursor protein-cleaving enzyme 2 (BACE2) is implicated in β-cell physiology, its clinical relevance in metabolic dysfunction remains unclear. Here, we investigated the clinical significance of circulating BACE2 in type 2 diabetes (T2D). Serum BACE2 was measured by enzyme-linked immunosorbent assay (ELISA) in 161 patients with T2D and 134 individuals with normal glucose tolerance. Circulating BACE2 was significantly elevated in T2D and independently associated with the presence of disease, with individuals in the highest quartile showing a 7.22-fold higher adjusted likelihood of T2D. BACE2 levels correlated positively with insulin resistance and dyslipidemia, with HbA1c emerging as the strongest determinant. Notably, BACE2 levels decreased following oral glucose loading in T2D, suggesting dynamic regulation in response to metabolic stimuli. Combining BACE2 with HOMA-IR markedly improved diagnostic performance (AUC 0.912 versus 0.712 for BACE2 alone). These findings identify circulating BACE2 as a biomarker reflecting chronic glycemic burden and metabolic stress in T2D.

## Highlights

• Circulating BACE2 is significantly elevated in type 2 diabetes
• BACE2 reflects chronic glycemic burden, with HbA1c as a key determinant
• Adding BACE2 to clinical models improves T2D diagnostic accuracy

## Graphical abstract

**Figure:**
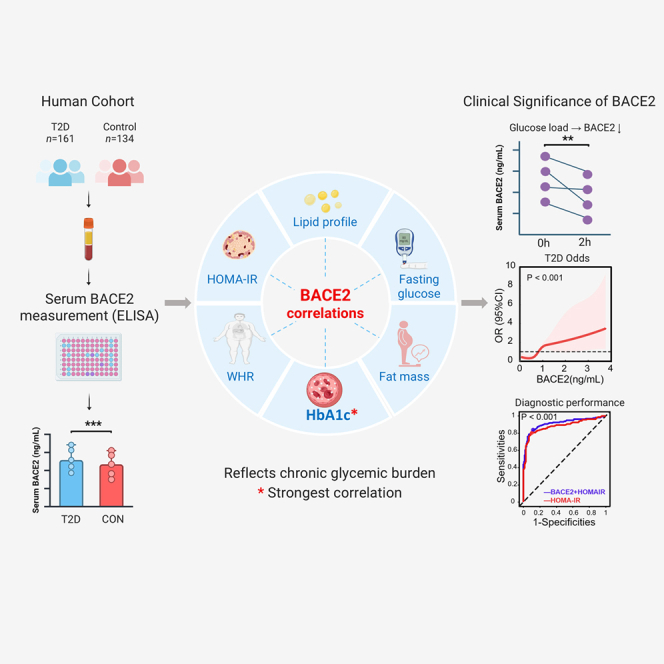

## Introduction

Type 2 diabetes (T2D) is a prevalent chronic metabolic disorder characterized by the interplay of insulin resistance and progressive pancreatic β-cell failure.^1^ In the early stages, β-cells compensate by increasing insulin secretion^2^; however, prolonged metabolic stress ultimately leads to β-cell dysfunction and hyperglycemia.^3^ Identifying biomarkers that reflect both glycemic burden and β-cell functional status is therefore critical for improving disease monitoring and therapeutic strategies.

β-site amyloid precursor protein-cleaving enzyme 2 (BACE2) is an aspartic protease predominantly expressed in pancreatic islets.^4^^,^^5^ It regulates β-cell function and glucose metabolism.^6^ Under physiological conditions, BACE2 supports insulin receptor trafficking and insulin secretion,^7^ whereas under chronic metabolic stress, increased BACE2 activity promotes β-cell dysfunction through proteolytic processing of key substrates.^8^^,^^9^^,^^10^ This stress-induced functional shift positions BACE2 as a molecular link in the progression from β-cell compensation to failure.

Despite strong preclinical evidence, translating these findings into a clinical context remains a significant gap. Prior human studies have focused primarily on static genetic variants of BACE2 associated with glycemic traits,^5^^,^^11^^,^^12^^,^^13^ leaving it unclear whether circulating BACE2 levels reflect metabolic state. This raises a question: could circulating BACE2 serve as a non-invasive, blood-based biomarker reflecting the transition from adaptive to maladaptive β-cell responses.

To address this gap, we investigated whether circulating BACE2 correlates with glycemic burden in T2D. In a Chinese cohort (*n* = 295; 161 T2D, 134 NGT), we quantified fasting and post-load BACE2 levels to examine associations with key metrics of metabolic dysfunction, T2D odds, and dynamic responses during an oral glucose tolerance test (OGTT). Additionally, we assessed its diagnostic accuracy alone and in combination with conventional metabolic indices. Therefore, this study aimed to evaluate the potential of circulating BACE2 as a clinical biomarker for glycemic burden and metabolic health in T2D.

## Results

### Clinical characteristics of the study population

No significant differences in age or sex distribution were observed between groups. Clinical and laboratory characteristics are detailed in Table S1. As anticipated, patients with T2D presented a less favorable metabolic profile than normal glucose tolerance (NGT) controls, characterized by significant hyperglycemia, hyperinsulinemia, dyslipidemia, and higher body mass index (BMI) and waist-to-hip ratio (WHR). Detailed clinical and laboratory parameters are summarized in Table 1.

**Table 1:** Summarized clinical, laboratory, and BACE2 characteristics of participants

Parameters	NGT (*n* = 134)	T2D (*n* = 161)	P
Age (years)	54.66 ± 8.29	55.43 ± 9.72	0.463
Gender (M/F)	70/64	83/78	0.907^a^
BMI (kg/m2)	23.97 ± 2.02	25.36 ± 2.41	<0.001
WHR	0.84 (0.78, 0.87)	0.91 (0.87, 0.97)	<0.001
SBP (mmHg)	116.0 (104.0, 124.3)	120.0 (112.5, 130)	0.002
DBP (mmHg)	74.50 (69.75, 82.00)	76.00 (70.5, 81.0)	0.147
FBG (mmol/L)	4.80 (4.48, 5.18)	8.00 (6.58, 9.49)	<0.001
2h-BG (mmol/L)	5.42 (4.65, 6.25)	18.65 (15.37, 22.57)	<0.001
FIns (mU/L)	6.91 (5.83, 8.17)	10.05 (6.77, 16.35)	<0.001
2h-Ins (mU/L)	34.63 (22.49, 52.48)	71.92 (41.57, 119.85)	<0.001
HbA1c (%)	5.30 (5.10, 5.50)	8.00 (7.00, 10.60)	<0.001
HOMA-IR	1.47 (1.16, 1.82)	3.71 (2.48, 6.05)	<0.001
HOMA-β	103.67 (74.80, 156.97)	52.97 (26.74, 79.59)	<0.001
TG (mmol/L)	1.07 (0.72, 1.67)	1.59 (1.06, 2.38)	<0.001
TC (mmol/L)	4.44 ± 1.17	4.63 ± 1.04	0.141
HDL-C (mmol/L)	1.20 (1.01, 1.44)	1.20 (1.01, 1.35)	0.326
LDL-C (mmol/L)	2.54 ± 0.96	2.60 ± 0.85	0.547
BACE2 (ng/mL)	0.66 (0.38, 0.91)	1.03 (0.69, 1.90)	<0.001
2h-BACE2 (ng/mL)	0.20 (0.06, 0.74)	0.94 (0.14, 2.13)	0.007

For continuous variables, we presented the data as mean and standard deviations (SDs) or medians (interquartile ranges). Categorical variables were presented as counts. Abbreviations: M/F, male/female; BMI, body mass index; WHR, waist-to-hip ratio; SBP, systolic blood pressure; DBP, diastolic blood pressure; FIns, fasting insulin; 2h-Ins, 2-h insulin; FBG, fasting blood glucose; 2h-BG, 2-h blood glucose; HOMA-IR, Homeostatic Model Assessment of Insulin Resistance; HbA1c, hemoglobin A1c; TG, triglycerides; TC, total cholesterol; HDL-C, high-density lipoprotein cholesterol; LDL-C, low-density lipoprotein cholesterol; 2h-BACE2, 2 h BACE2; a, chi-square test. All patients with T2D were newly diagnosed and treatment-naïve; detailed lifestyle factors were not comprehensively captured.

### Comparison of serum BACE2 levels and dynamic response to OGTT

**Figure 1:**
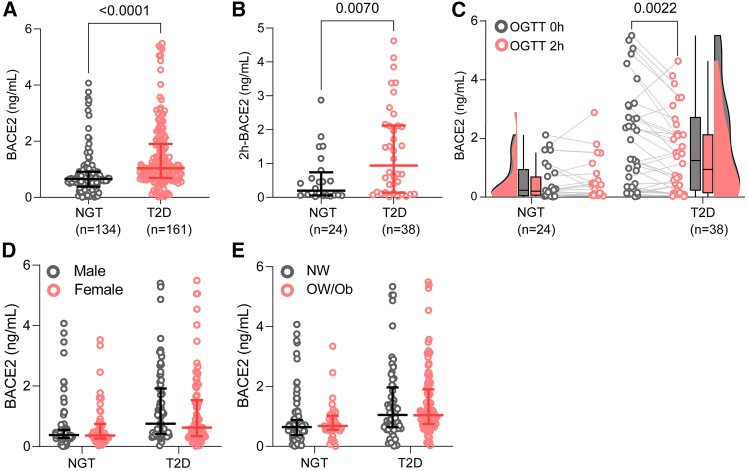
Comparison of serum BACE2 levels, dynamic responses, and subgroup stratifications between NGT and T2D participants (A) Fasting serum BACE2 concentrations in the total cohort (Mann-Whitney U test, NGT, *n* = 134; T2D, *n* = 161). (B) Serum BACE2 concentrations at 2 h during an oral glucose tolerance test (OGTT) in a subgroup (Mann-Whitney U test, NGT, *n* = 24; T2D, *n* = 38). (C) Dynamic changes in serum BACE2 levels during the OGTT at 0 h and 2 h (Wilcoxon signed-rank test, NGT, *n* = 24; T2D, *n* = 38). (D and E) Serum BACE2 concentrations stratified by gender and BMI. Sample sizes for NGT (male, *n* = 70, female, *n* = 64 (D); NW, normal weight, *n* = 99, OW/Ob, overweight/obese, *n* = 35 (E)) and T2D (male, *n* = 83, female, *n* = 78 (D); NW, *n* = 64, OW/Ob, *n* = 97 (E)). Data are analyzed by the Mann-Whitney U-test and presented as scatterplots with median and IQR.

Serum BACE2 concentrations were significantly elevated in the T2D group compared to the NGT group (median: 1.03 [0.69–1.90] vs. 0.66 [0.38–0.91] ng/mL, *p* < 0.001; Figure 1A). This difference remained significant at the 2-h time point during the OGTT (0.94 [0.14–2.13] vs. 0.20 [0.06–0.74] ng/mL, *p* = 0.007; Figure 1B). In a subgroup analysis of the dynamic response, BACE2 levels in patients with T2D exhibited a significant acute decline 2 h after the glucose challenge (*p* = 0.002), whereas no significant change was observed in NGT individuals (Figure 1C). Notably, baseline BACE2 concentrations did not differ significantly when stratified by gender or BMI categories (Figures 1D and 1E), although a weak positive correlation between BACE2 and BMI was observed in the overall cohort (r = 0.187, *p* = 0.001).

### Logistic regression analysis revealed an independent association between circulating BACE2 and T2D

Univariate and multivariable logistic regression analyses were performed to assess the association between BACE2 and the presence of T2D (Table 2). In the unadjusted model, BACE2, BMI, and total cholesterol (TC) were significantly associated with increased odds of T2D (all *p* < 0.001). After adjustment for potential confounders (age, gender, BMI, and lipid profiles), BACE2 remained a robust independent indicator. Specifically, each 1 ng/mL increase in serum BACE2 was associated with 2.10-fold higher odds of T2D (adjusted OR = 2.098, 95% CI: 1.54–2.98; *p* < 0.001).

**Table 2:** Univariate and multivariable logistic regression analyses of circulating BACE2 and metabolic factors associated with T2D

Variables	Univariate					Multivariate				
	β	S.E	Z	P	OR (95%CI)	β	S.E	Z	P	OR (95%CI)
BACE2	0.746	0.165	4.533	<0.001	2.109(1.560–2.981)	0.741	0.167	4.441	<0.001	2.098(1.543–2.976)
Male					1.00 (Reference)					1.00 (Reference)
Female	0.027	0.234	0.117	0.907	1.028 (0.650–1.627)	0.124	0.269	0.461	0.645	1.132 (0.668–1.924)
Age	0.009	0.013	0.725	0.468	1.009 (0.984–1.035)	0.031	0.015	2.022	0.043	1.032 (1.001–1.064)
BMI	0.285	0.058	4.897	<0.001	1.330 (1.190–1.496)	0.272	0.062	4.370	<0.001	1.313 (1.166–1.490)
TG	0.159	0.108	1.468	0.142	1.172 (0.950–1.455)	0.411	0.292	1.411	0.158	1.509 (0.846–2.692)
TC	0.521	0.136	3.839	<0.001	1.684 (1.309–2.230)	0.468	0.156	3.008	0.003	1.597 (1.195–2.214)
HDL	−0.131	0.328	−0.400	0.689	0.877 (0.454–1.678)	−0.295	0.463	−0.638	0.524	0.745 (0.306–1.907)
LDL	0.075	0.130	0.575	0.565	1.078 (0.836–1.395)	−0.576	0.341	−1.688	0.091	0.562 (0.282–1.087)

Abbreviations: BMI, body mass index; TG, triglycerides; TC, total cholesterol; HDL, high-density lipoprotein cholesterol; LDL, low-density lipoprotein cholesterol; β, regression coefficient; S.E., standard error; Z, Z-statistic; OR, odds ratio; CI, confidence interval. Notes: The multivariable model included all variables listed in the univariate analysis to identify independent risk factors.

### Correlation between serum BACE2 levels and metabolic parameters

BACE2 levels were positively correlated with multiple markers of metabolic dysregulation, including fasting blood glucose (FBG), HbA1c, HOMA-IR, and adverse lipid profiles in all subjects (Table 3). Among these, HbA1c showed the strongest correlation (r = 0.371, *p* < 0.001). In a multivariable linear regression model, HbA1c emerged as the primary independent factor associated with BACE2 levels (β = 0.303, *p* < 0.001). Linear regression further highlighted this relationship, indicating that each 1 ng/mL increase in BACE2 corresponded to a 0.711% increase in HbA1c, underscoring its link to chronic glycemic burden.

**Table 3:** Correlation and regression analyses of circulating BACE2 in all subjects

Variables	BACE2		BACE2 (Gender-adjusted)		Multivariate	
	r	P	r	P	β	P
Age	−0.014	0.816	−0.002	0.968	–	–
BMI	0.187	0.001	0.059	0.314	–	–
WHR	0.174	0.003	0.192	0.001	–	–
SBP	0.014	0.807	−0.053	0.363	–	–
DBP	−0.005	0.936	−0.008	0.894	–	–
FIns	0.124	0.033	0.011	0.875	–	–
FBG	0.300	<0.001	0.189	0.001	–	–
HOMA-IR	0.216	<0.001	0.078	0.181	–	–
HOMA-β	−0.239	<0.001	−0.132	0.023	–	–
HbA1c	0.371	<0.001	0.301	<0.001	0.303	<0.001
TG	0.122	0.037	0.020	0.733	–	–
TC	0.103	0.076	0.018	0.753	–	–
HDL-C	0.035	0.545	0.130	0.026	–	–
LDL-C	0.109	0.062	−0.006	0.917	–	–

Abbreviations: BMI, body mass index; WHR, waist-to-hip ratio; SBP, systolic blood pressure; DBP, diastolic blood pressure; FIns, fasting insulin; FBG, fasting blood glucose; HOMA-IR, Homeostatic Model Assessment of Insulin Resistance; HbA1c, hemoglobin A1c; TG, triglycerides; TC, total cholesterol; HDL-C, high-density lipoprotein cholesterol; LDL-C, low-density lipoprotein cholesterol. Notes: r, Spearman’s correlation coefficient; β, standardized regression coefficient. BACE2 (Gender-adjusted) values represent partial correlation coefficients. The multivariate linear regression model (stepwise) initially included all parameters significant in the univariate analysis.

### Quartile-based odds and performance of BACE2 as a complementary biomarker for T2D

**Figure 2:**
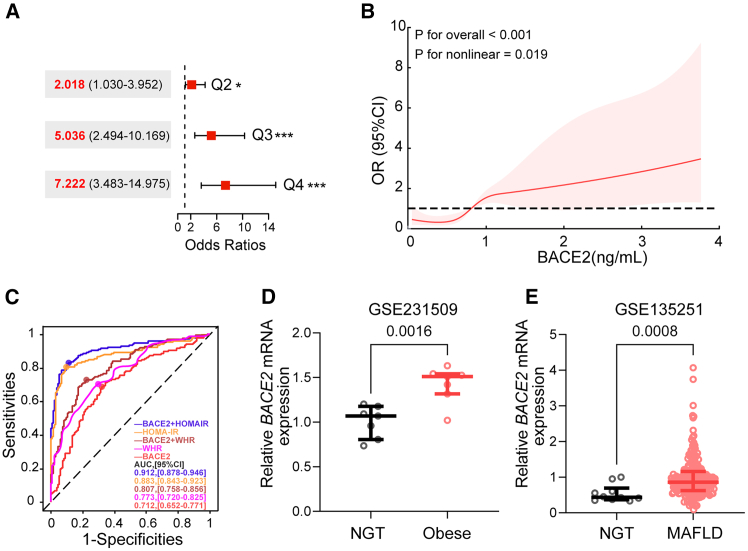
Association of circulating BACE2 with T2D odds and tissue metabolic stress (A) Odds ratios (ORs) and 95% confidence intervals (CIs) for T2D across serum BACE2 quartiles (Q1-Q4) calculated using multivariable logistic regression (*n* = 295). ∗*p* < 0.05, ∗∗∗*p* < 0.001 vs. Q1. (B) Restricted cubic spline (RCS) analysis depicts the non-linear association between serum BACE2 levels and the odds ratio (OR) for T2D (*n* = 295, logistic regression model). The solid red line represents the adjusted OR, and the shaded area indicates the 95% CI. The dashed line represents an OR of 1.0. (C) Receiver operating characteristic (ROC) curves for BACE2 alone and in combination with HOMA-IR or BMI for T2D discrimination, along with their diagnostic performance (AUC, cutoff, sensitivity, specificity): BACE2 (0.712, 0.771, 68.9%, 67.9%), BMI (0.690, 24.78, 63.4%, 73.1%), WHR (0.773, 0.865, 70.2%, 70.1%), HOMA-IR (0.883, 2.24, 80.7%, 90.3%), and BACE2+HOMA-IR model (0.912, 0.474, 83.2%, 88.8%). (*n* = 295). (D and E) Validation of BACE2 mRNA expression in metabolic tissues from public datasets: skeletal muscle in obese individuals (GSE231509; Mann-Whitney U test, NGT, *n* = 7; Obese, *n* = 7) (D) and liver in patients with non-alcoholic fatty liver disease (NAFLD) (GSE135251; Mann-Whitney U test, NGT, *n* = 10, MAFLD, *n* = 206) (E). AUC, area under the curve; HOMA-IR, homeostasis model assessment of insulin resistance. Data are presented as scatterplots with median and IQR.

When participants were stratified into quartiles based on BACE2 concentration, the odds of T2D increased progressively across quartiles. Compared to the lowest quartile (Q1), individuals in Q3 (OR: 5.04) and Q4 (OR: 7.22; 95% CI: 3.48–14.98) faced significantly higher odds of T2D (both *p* < 0.001; Figure 2A). Restricted cubic spline (RCS) analysis revealed a significant non-linear relationship between BACE2 levels and the odds of T2D (P for overall <0.001, P for non-linearity = 0.019; Figure 2B). The odds ratio for T2D began to increase sharply as BACE2 levels rose beyond approximately 0.8 ng/mL. After reaching about 1.5 ng/mL, the odds continued to rise but at a more moderate pace at higher concentrations.

Receiver operating characteristic (ROC) analysis showed that BACE2 yielded an AUC of 0.712 (95% CI: 0.652–0.771), with an optimal cutoff of 0.77 ng/mL (sensitivity 68.9%, specificity 67.9%). This performance was comparable to BMI (AUC = 0.690) and WHR (AUC = 0.773), but lower than HOMA-IR (AUC = 0.883, *p* < 0.001; Figure 2C).

Combining BACE2 with HOMA-IR significantly improved discrimination, achieving the highest AUC of 0.912 (*p* < 0.001 vs. HOMA-IR alone; Figure 2C). Furthermore, adding BACE2 to a base clinical model adjusted for age, sex, BMI, and lipid profiles resulted in a modest but significant increase in AUC (from 0.732 to 0.771, *p* = 0.045; Figure S1A). Collectively, these findings suggest that BACE2 provides incremental discriminatory value beyond established clinical covariates.

### Upregulated BACE2 transcript levels in metabolic tissues

To explore potential tissue sources of circulating BACE2, we analyzed public transcriptomic datasets. BACE2 mRNA expression was significantly elevated in the skeletal muscle of obese individuals (*p* = 0.002, Figure 2D) and in the liver tissue of patients with non-alcoholic fatty liver disease (NAFLD) (*p* < 0.001, Figure 2E) compared to healthy controls.^14^^,^^15^ These findings suggest that insulin-sensitive organs under metabolic stress may contribute to the elevated systemic BACE2 levels observed in T2D.

## Discussion

This study provides the first clinical evidence that circulating BACE2 is elevated in T2D and independently associated with chronic glycemic burden. Individuals in the highest serum BACE2 quartile exhibited a markedly increased odds of T2D (OR: 7.22; 95% CI: 3.48–14.98), and this association remained significant after adjustment for age, sex, BMI, and lipid profiles. Rather than serving as a primary diagnostic marker, these findings suggest that circulating BACE2 reflects underlying metabolic stress associated with sustained dysglycemia and may provide complementary information beyond conventional clinical indices. The contribution of insulin-sensitive tissues, including the pancreas, liver, and skeletal muscle, may underlie the observed elevation of circulating BACE2 in T2D.

A critical question is whether the elevation of circulating BACE2 merely reflects hyperglycemia. Several lines of evidence argue against this interpretation. First, circulating BACE2 showed a stronger independent association with HbA1c than with FBG. Second, mediation analysis indicated that fasting glucose did not significantly mediate the association between BACE2 and T2D odds (ACME, *p* = 0.12), with the direct effect accounting for the majority of the total effect (Figure S1B). Collectively, these findings suggest that circulating BACE2 reflects cumulative, chronic metabolic stress rather than short-term glycemic fluctuations. This “chronic stress” hypothesis is supported by preclinical evidence: while physiological BACE2 expression maintains insulin signaling and β-cell function,^7^^,^^16^ sustained glucotoxicity upregulates BACE2 via oxidative stress pathways.^17^^,^^18^ Under the persistent glucolipotoxicity characteristic of T2D, as reflected by elevated HbA1c, this adaptation may transition into a maladaptive phase. Excessive BACE2 activity could shift substrate preference: while BACE2 physiologically cleaves TMEM27 to promote β-cell proliferation, pathological upregulation may favor the cleavage of pro-IAPP into amyloidogenic peptides, thereby accelerating islet amyloid deposition and β-cell loss.^8^^,^^18^^,^^19^^,^^20^ Accordingly, the elevated circulating BACE2 observed in our T2D cohort likely signifies proteostatic stress and exhaustion of β-cell compensatory capacity.

Beyond its basal elevation, the acute suppression of serum BACE2 during the OGTT provides novel insight into its dynamic regulation. The rapid decline post-glucose challenge suggests that BACE2 release is actively modulated by nutrient sensing. Consistent with this, emerging evidence identifies BACE2 as a sophisticated nutrient sensor, with its expression finely tuned by extracellular nutrient availability to regulate surface transporter abundance and lipid droplet dynamics.^21^ The more pronounced reduction in the T2D group may reflect a maladaptive or exaggerated regulatory response under IR conditions, mirroring the broader loss of metabolic flexibility in T2D.^2^ This contrasts with observations in gestational dysglycemia, where BACE2 variants primarily affect fasting levels,^5^^,^^11^ implying a distinction between the genetic determinants of baseline levels and acute dynamic responsiveness.

Beyond glucose sensing, circulating BACE2 was also significantly associated with dyslipidemia, evidenced by positive correlations with triglycerides, TC, and LDL-C. Mechanistically, hepatic BACE2 has been shown to cooperate with ENO1 to cleave the LDL receptor, impairing cholesterol clearance and potentially triggering compensatory synthesis.^22^ Furthermore, BACE2, assisted by the etraspanin CD63, regulates lipid homeostasis by mediating the extracellular shedding of transporters such as LDLR and CD36, thereby fine-tuning lipid uptake and maintaining lipid droplet stability.^21^ Analysis of public transcriptomic datasets corroborated this multi-organ role, showing upregulated BACE2 expression in the skeletal muscle of obese individuals and the livers of patients with NAFLD.^14^^,^^15^ Together with its high basal expression in β-cells,^4^ these findings suggest that the circulating BACE2 pool represents an integrated signal of multi-organ metabolic stress, linking glucose and lipid dysregulation.

From a clinical perspective, stratified analysis revealed a non-linear relationship between BACE2 and BMI. The association was strongest in the intermediate BMI quartile (Q2) but attenuated in higher quartiles (Q3-Q4), suggesting a potential saturation effect. This pattern implies that BACE2 may be particularly responsive during the metabolic transition from normal weight to overweight, consistent with reports of early β-cell dysfunction in BACE2-deficient mice under obesogenic conditions.^13^ While BACE2 alone demonstrated moderate diagnostic utility (AUC = 0.712), its performance was substantially enhanced when combined with BMI (AUC = 0.824) or HOMA-IR (AUC = 0.912), underscoring its value as a complementary biomarker that captures specific dimensions of subclinical β-cell stress.

Although BACE2 is a transmembrane protease, its detection in serum parallels established paradigms in protease biology, such as BACE1 in neurodegenerative contexts^23^^,^^24^^,^^25^^,^^26^^,^^27^ or Corin in heart failure.^28^ Current evidence suggests that BACE2 is released into the circulation via vesicular secretion, a process frequently heightened under conditions of cellular stress. The tetraspanin CD63 is essential for trafficking and positioning BACE2 within specific vesicular compartments, such as multivesicular bodies, thereby facilitating substrate cleavage.^21^ Given that CD63 is a canonical exosomal marker enriched in intraluminal vesicles,^29^^,^^30^ our enzyme-linked immunosorbent assay (ELISA) specifically quantifies the shed, soluble extracellular domain of BACE2 (amino acids 63–466), which contains the catalytically active site. This supports the hypothesis that circulating BACE2 may function not merely as a passive signal, but as an active protease in the systemic circulation, potentially contributing to T2D pathophysiology by cleaving substrates in the bloodstream or at distant tissues.

In conclusion, this study provides clinical evidence that elevated circulating BACE2 is independently associated with chronic metabolic stress in T2D, including hyperglycemia, dyslipidemia, and IR. BACE2 may serve as a promising biomarker reflecting a state of glucolipotoxic burden, potentially offering complementary information beyond conventional clinical indices. Future longitudinal and mechanistic studies are essential to validate the clinical utility of BACE2 and to explore therapeutic potential in metabolic disorders.

### Limitations of the study

This study has several limitations. First, its cross-sectional design precludes causal inference. Second, the small OGTT subgroup limits power for dynamic analyses. Third, the absence of adult patients with T1D limits the generalizability to autoimmune diabetes. Fourth, the ELISA detects only the soluble extracellular domain, potentially missing full-length protein or localized activity. Finally, incomplete lifestyle factors may introduce residual confounding. Although BMI and lipid profiles were included as surrogate metabolic indicators, they cannot fully capture lifestyle-related heterogeneity. Future longitudinal and mechanistic studies are warranted to clarify the pathogenic role of BACE2 in T2D.

## Resource availability

### Lead contact

Requests for further information should be directed to and will be fulfilled by the lead contact, Dr. Shiqiao Zhao (qiao.830@163.com).

### Materials availability

This study did not generate new or unique reagents.

### Data and code availability

#### Data

All data reported in this paper will be shared by the lead contact upon request. Source data have been deposited at Mendeley Data: https://doi.org/10.17632/h72yzh3wkd.3.

Publicly available datasets used in this study include transcriptomic datasets from NCBI GEO: GSE231509 and GSE135251.

#### Code

This paper does not report the original code.

#### Other items

Any additional information required to reanalyze the data reported in this paper are available from the lead contact upon request.

## Acknowledgments

This work was partially supported by the 10.13039/501100005230Natural Science Foundation of Chongqing (grant no. cstc2021jcyj-msxmX0519), the Basic Research Project of the Key Laboratory of Childhood Developmental Disorders Research, and Ministry of Education (grant no. YBRP-202120). We are thankful for the participation of patients with T2D and NGT individuals.

## Author contributions

Conceptualization, B.L. and S.Q.Z.; methodology, B.L., Z.X.L., and Z.Y.C.; investigation, B.L., Z.X.L., and Z.Y.C.; writing – original draft, B.L., and H.W.; writing—review and editing, H.W., and S.Q.Z.; funding acquisition, B.L., H.W., and S.Q.Z.; resources, B.L., Z.X.L., and Z.Y.C.; supervision, H.W., and S.Q.Z.

## Declaration of interests

The authors declare no competing interests.

## STAR★Methods

### Key resources table

**Table:** 

REAGENT or RESOURCE	SOURCE	IDENTIFIER
**Critical commercial assays**		

Human BACE2 ELISA KIT	Novus Biologicals	Cat # NBP2-66745

**Deposited data**		

Skeletal muscle from obese and lean individuals	Gene Expression Omnibus	GSE231509
Liver tissue from NAFLD patients and healthy controls	Gene Expression Omnibus	GSE135251
Clinical metadata from this study	Mendeley Data	https://doi.org/10.17632/h72yzh3wkd.3

**Biological samples**		

Serum samples from T2D and NGT individuals	Chongqing Traditional Chinese Medicine Hospital	The Ethics Committee of Chongqing Traditional Chinese Medicine Hospital (No. 2025-KYHY-5)

**Software and algorithms**		

GraphPad Prism 9	GraphPad Software	https://www.graphpad.com/
R project	4.2.2	https://cran.r-project.org/

### Experimental model and study participant details

A total of 295 participants of Han Chinese were recruited for this cross-sectional study at Chongqing Traditional Chinese Medicine Hospital between January and December 2025. Based on the results of a 75 g oral glucose tolerance test (OGTT) and the 1999 WHO diagnostic criteria for diabetes mellitus,^31^ participants were classified into a T2D group (n = 161; 83 males and 78 females; age range: 29–77 years) and a NGT control group (n = 134; 70 males and 64 females; age range: 28–72 years). All T2D patients were newly diagnosed and had not received any glucose-lowering medications. The potential influence of sex on the primary outcome (circulating BACE2 concentrations) was evaluated, and no significant associations were observed.

Comprehensive medical and family histories were obtained, and all individuals underwent standard clinical assessments under routine medical conditions. Exclusion criteria included: (1) other diabetes types (T1D, gestational diabetes mellitus); (2) significant renal or thyroid dysfunction; (3) acute or chronic infections; (4) severe cardiovascular or cerebrovascular diseases; (5) malignancies; and (6) psychiatric disorders.

This study was conducted in accordance with the Declaration of Helsinki and was approved by the Ethics Committee of Chongqing Traditional Chinese Medicine Hospital (No. 2025-KYHY-5). Written informed consent was obtained from all participants prior to inclusion. This study exclusively involved human participants, and no animal or cell-based experiments were conducted.

### Method details

#### Clinical and biochemical measurements

After a 10-12 h overnight fast, anthropometric and biochemical parameters were assessed. Height, weight, and waist circumference were measured to calculate body mass index (BMI) and waist-to-hip ratio (WHR). Fasting blood samples were centrifuged at 3500 × g for 10 min at 4°C; serum was aliquoted and stored at -80°C until analysis. Fasting blood glucose (FBG) was measured using the glucose oxidase method; fasting insulin (FIns) by electrochemiluminescence immunoassay (Roche E411, Switzerland); and glycated hemoglobin (HbA1c) by high-performance liquid chromatography (HPLC; Bio-Rad, USA). Serum lipids, including total cholesterol (TC), high-density lipoprotein cholesterol (HDL-C), low-density lipoprotein cholesterol (LDL-C), and triglycerides (TG), were quantified with enzymatic colorimetric assays (Siemens Advia 2400, Germany). Insulin resistance and β-cell function were estimated using the homeostasis model assessment (HOMA): HOMA-IR = [FBG (mmol/L) × FIns (mU/L)]/22.5; HOMA-β = [20 × FIns (mU/L)]/[FBG (mmol/L) - 3.5].

#### Oral glucose tolerance test (OGTT) and serum BACE2 measurement

All participants underwent a standard 75-g OGTT after an overnight fast. Fasting serum BACE2 levels were quantified for the entire cohort (n = 295), while 2-h post-load concentrations were measured in a random subgroup (n = 62; 24 NGT, 38 T2D) to assess acute nutrient-responsive dynamics. Serum BACE2 was measured in duplicate using a commercial sandwich ELISA kit (Novus Biologicals, USA; Cat. No. NBP2-66745, specifically quantified the soluble extracellular domain of BACE2). Briefly, 100 μL of undiluted serum was incubated in pre-coated wells for 90 min at 37°C followed by sequential incubation with biotinylated detection antibody for 1 hour and HRP conjugate for 30 min, both at 37°C. After the final wash and a 15-min colorimetric reaction, the optical density was measured at 450 nm. Concentrations were calculated using a four-parameter logistic regression model based on a 7-point standard curve.

#### Public transcriptomic data analysis

To explore potential tissue sources of circulating BACE2, we analyzed two publicly available human transcriptomic datasets from the Gene Expression Omnibus (GEO): GSE231509 (skeletal muscle from obese vs. lean individuals) and GSE135251 (liver tissue from NAFLD patients vs. healthy controls). Data were normalized using the Robust Multi-array Average (RMA) method, and differential expression was assessed using the limma package, applying false discovery rate (FDR) correction (Benjamini-Hochberg method) for multiple testing. An adjusted P-value (FDR) < 0.05 was considered statistically significant.

### Quantification and statistical analysis

Statistical analyses were conducted using GraphPad Prism (v9.5.0) and R (v4.2.1). Continuous variables are presented as means ± SD or medians with IQR; categorical variables as frequencies and percentages. Normality was tested by Kolmogorov-Smirnov test, and variance homogeneity by Levene’s test. Two-group comparisons used Student’s t-test (equal variances), Welch’s t-test (unequal variances), or Mann-Whitney U test (non-normal distribution); categorical data by χ^2^ test. Multivariable linear/logistic regression models (for BACE2 determinants and T2D odds, respectively) were built with variables selected based on the metabolic characteristics of T2D, adjusted for age, gender, BMI, and lipid profiles.^32^ Causal mediation analysis (R “Mediation” package) used linear regression for mediator (FBG) and logistic regression for outcome (T2D), adjusted for age, sex, and BMI; ACME and ADE were estimated with 1,000 bootstraps, assuming sequential ignorability. Non-linear associations were modeled by RCS (5 knots). Diagnostic performance was evaluated using ROC curves. The optimal cutoff values were determined by maximizing the Youden index (sensitivity + specificity-1). Statistical comparisons between different AUCs were performed using DeLong’s test. Two-tailed *P* < 0.05 was significant.

## Supplemental information

Document S1. Figure S1

Table S1. Detailed clinical, laboratory, and BACE2 characteristics of participants
