# Supplementary material for: Elevated circulating BACE2 captures chronic glycemic burden and enhances the clinical identification of type 2 diabetes
Source: iScience. 2026 May 22;29(6):115988. doi: 10.1016/j.isci.2026.115988 (PMC13217922; doi:10.1016/j.isci.2026.115988)
Supplement: Document S1. Figure S1 [file mmc1.pdf]

iScience, Volume *29*

Supplemental information

Elevated circulating BACE2 captures chronic glycemic burden and enhances the clinical identification of type 2 diabetes

Bo Li, Zhixing Luo, Zhongyu Chen, Han Wang, and Shiqiao Zhao

Supplementary Figure S1

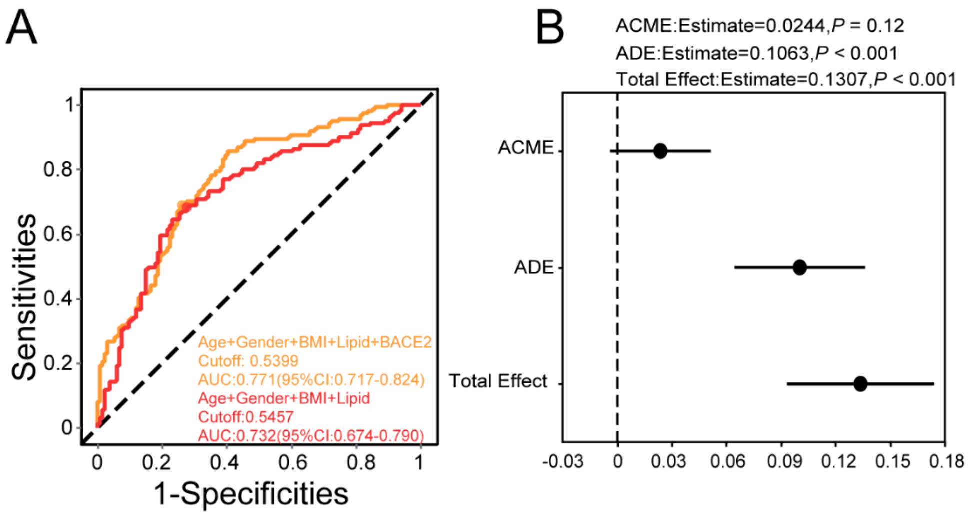

Figure S1. Diagnostic performance and hyperglycemia independence of circulating BACE2 in T2D

(A) ROC curves showing the incremental gain in T2D discrimination when BACE2 is added to the base model (age, sex, BMI, and lipids). Comparisons of AUCs were performed using DeLong's test (n = 295).

(B) Mediation analysis assessing whether FBG mediates the association between BACE2 and the odds of T2D, adjusted for age, sex, and BMI. The average direct effect (ADE) was significant (estimate = 0.106, P < 0.001), whereas the average causal mediation effect (ACME) through FBG was not significant (estimate = 0.024, P = 0.12). Statistical significance was estimated using 1000 bootstrap resamples (n = 295).
